# Assessment of seasonality in soil-transmitted helminth infections across 14 schools in Jimma Town, Ethiopia

**DOI:** 10.11604/pamj.2019.32.6.16085

**Published:** 2019-01-04

**Authors:** Zeleke Mekonnen, Mestawet Getachew, Johannes Bogers, Jozef Vercruysse, Bruno Levecke

**Affiliations:** 1 School of Medical Laboratory Sciences, Institute of Health, Jimma University, Jimma, Ethiopia; 2 School of Pharmacy, Institute of Health, Jimma University, Jimma, Ethiopia; 3 Applied Molecular Biology Research (AMBIOR) Group, Antwerp University, Antwerp, Belgium; 4 Department of Virology, Parasitology and Immunology, University of Ghent, Belgium

**Keywords:** Soil-transmitted helminths, prevalence, intensity, seasonality, Jimma, Ethiopia

## Abstract

**Introduction:**

Up to date, the frequency of preventive chemotherapy based on the prevalence is the only strategy in the control programmes of soil-transmitted helminths (STHs). However, prevalence of STHs may be affected by climatic and/or seasonal changes, particularly when these are important determinants of transmission of STH infections. Our objective was to describe the prevalence and infection intensity and seasonal variation (mainly dry vs rainy season) of any STHs among school age children.

**Methods:**

Assessment of infection intensity and prevalence of STHs was carried out during dry season (February-March, 2012) and end of rainy season (September-October, 2012) across 14 primary schools in Jimma Town, Jimma, Ethiopia. A total of 1,680 school children (840 in each season) were included. All stool samples were processed by the McMaster egg counting method. Odds of infection and intensity was performed to assess any differences in prevalence and infection intensity between the schools and the two seasons. The pooled odd ratio and their 95% confidence interval was also computed and presented using the "metafor" package of the statistical software R. The level of significance was declared at p < 0.05.

**Results:**

Infections with any STH were observed in 824/1,680 (49.0%) subjects. *T. trichiura* was the most prevalent (35.5%), followed by *A. lumbricoides* (23.4%) and hookworms (9.9%). Among the schools there were a huge variation in prevalence, ranging from 16.7% to 68.3% for any STH, 6.7% to 39.2% for *A. lumbricoides*, 10.8% to 55.0% for *T. trichiura* and 0 % to 28.3% for hookworms. A significant difference in prevalence (for *T. trichiura*) and in infection intensity (for *A. lumbricoides* and *T. trichiura*) across seasons was observed. Generally, STH infections were more prevalent in the dry season (52.4%) compared to the rainy season (45.7%) and as well intensity of all three STH infections was higher in the dry season.

**Conclusion:**

Our data suggested that there were huge variation in STH prevalence among schools and a significant difference in infection intensity and prevalence across seasons. This in turn might limits how national governments and international organizations define and target resources to combat the disease burden due to STH infection. Long term studies are needed to confirm the influence of seasonal factors and related ecological, environmental and socio-economic factors.

## Introduction

Over five billion people of the world are at risk of infection from soil-transmitted helminths (STHs), caused by the roundworm *Ascaris lumbricoides*, the whipworm *Trichuris trichiura* and the two hookworm species *Ancylostoma duodenale* and *Necator americanus* [1]. It is estimated that in 2010 approximately 1.4 billion people were infected worldwide, accounting for 20% of the disability-adjusted life years caused by NTDs [1, 2]. The highest prevalence data are reported in tropical and subtropical regions from Sub-Saharan Africa, the Americas, China and East Asia [3-5]. Up to date, the frequency of preventive chemotherapy (PC) is based on the prevalence is the only strategy in the control programmes of STHs. Based on the STHs prevalence, either Albendazole or Mebendazole drugs being administered annually when the overall STHs prevalence is at least 20% but less than 50% or bi-annually when the prevalence exceeds 50% [6-8]. However, this STH prevalence may be affected by climatic and/or seasonal changes, particularly when these are important determinants of transmission of STH infections. Adequate moisture and warm temperature are essential for egg/larval development in the soil [1, 8, 9]. For example, eggs of *A. lumbricoides* and *T. trichiura* will not embryonate at low humidity, whereas higher humidity is associated with faster development of eggs [10, 11] whereby in turn, these differences in development and survival will affect and influence parasite establishment in the human host and, hence, infection levels.

In wet seasons, pre-parasitic stages of worms might survive in the environment that favours and increases in transmission. This is in contrast with dry climatic conditions, which kills deposited infective stages on the soil surface making the dynamics for transmission to decline [9]. Previous studies reported that high STHs infection was detected in rainy season compared to summer season and comparatively lower incidence of STH was noticed in winter season [12, 13]. Overall, many epidemiological surveys conducted in different countries revealed correlation between the prevalence of parasitic infections including STHs and seasons of the years [9, 12-14]. It has been described that understanding of the seasonal changes of STHs is important for appropriately time-scheduling of PC, thereby maximizing the cost-effectiveness of these programmes and to support public health decision-making to launch PC programme [3, 7, 15]. For instance determining prevalence at times of the year when there is a low transmission of STHs might underestimate the actual prevalence of infections and can influence the implementation of prevalence based large-scale PC interventions. Like most other African regions, in Ethiopia in general and in Jimma (our study area) in particular, there is inadequate reliable information concerning the prevalence of STH infections across different seasons. Therefore, our main objective was to describe the prevalence and infection intensity of any STHs in general and the three species in particular in two different seasons (dry vs rainy season) across 14 primary schools in Jimma Town.

## Methods

**Figure 1:**
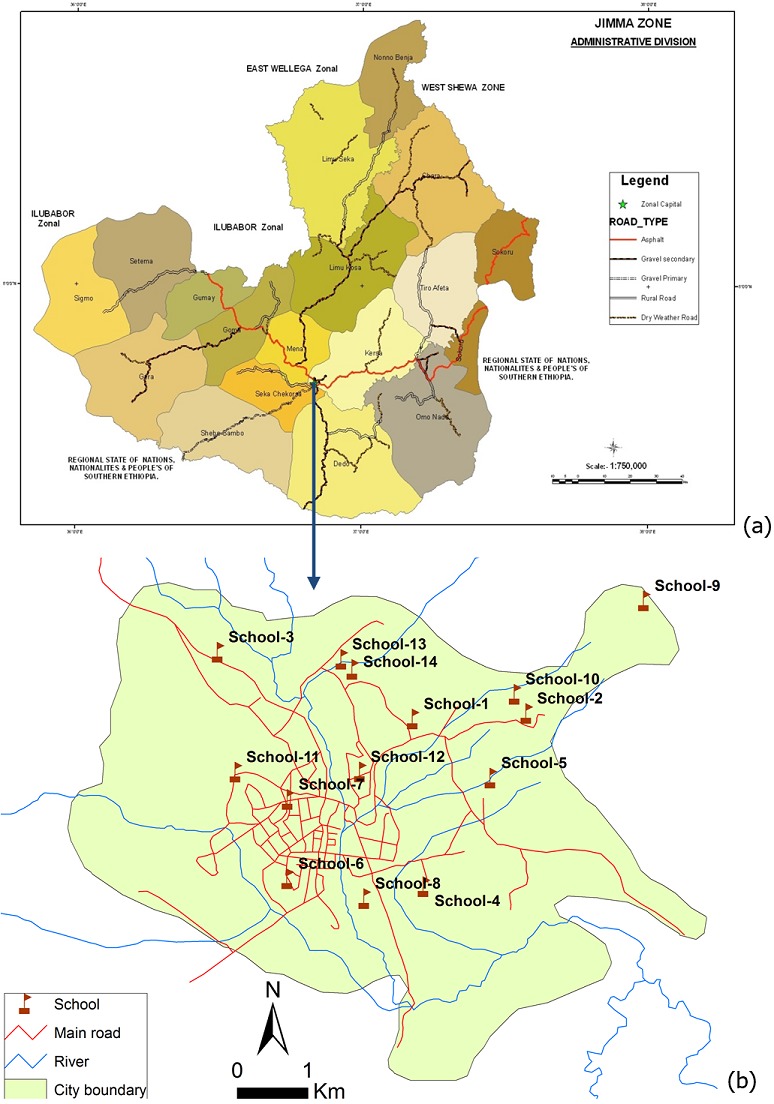
(A) map of Jimma zone; (B) map of Jimma city indicating specific study sites (=14 Schools) with in the Jimma Town, Capital of Jimma Zone

**Study sites and population:** The study was conducted in Jimma Town, Ethiopia, located approximately 352 km southwest of the capital, Addis Ababa. Jimma Town is situated at a latitude and longitude of 7° 40' N 36° 50' E and is characterized by a semi-arid type climate with an average annual rainfall of 800-2,500 mm. It is situated 1,720-2,010m above sea level and lies in the climatic zone locally known as Woina-dega, which is considered ideal for agriculture as well as human settlement. Like most sub-tropical (Woina-dega) region of Ethiopia, Jimma Zone is known to have the four typical kind of seasons in a year as spring (September-November (end of rainy season)), winter (December-February (dry season)), autumn (March-May (beginning of rainy season) and (summer (June-August (rainy season). Of note, the term winter as it is used here may be misleading and hence it worth's for the reader to note it in Ethiopian context that this term will not represent the characteristics of winter weather elsewhere. However, the town is generally characterized by warm climate with mean daily temperature of 19°C and ranges from 12 to 30°C. Maximum precipitation and heavy rain occurs during the three months period, June to August, with minimum rainfall in December and January, making practically Jimma to be recognized by two major seasons as dry season (December-May) and rainy or wet season (June-November). Our study focused on school children from age 5 to age 18, across all eight grades (grade 1 to 8). In total, there were 24 primary schools during the study fiscal year hosting a total of 23,492 children of all age groups of interest (Figure 1, A) shows the map of Jimma Zone and Figure 1, B) shows schools studied in Jimma Town, the capital of the zone. The female/male ratio across the different schools was approximately 1:1 (Report document 2011/2012 of Jimma Education Bureau). STH infections have been documented in Jimma Town, but at the time of this study no PC program to control STHs in school age children (SAC) has been implemented.

**Figure 2:**
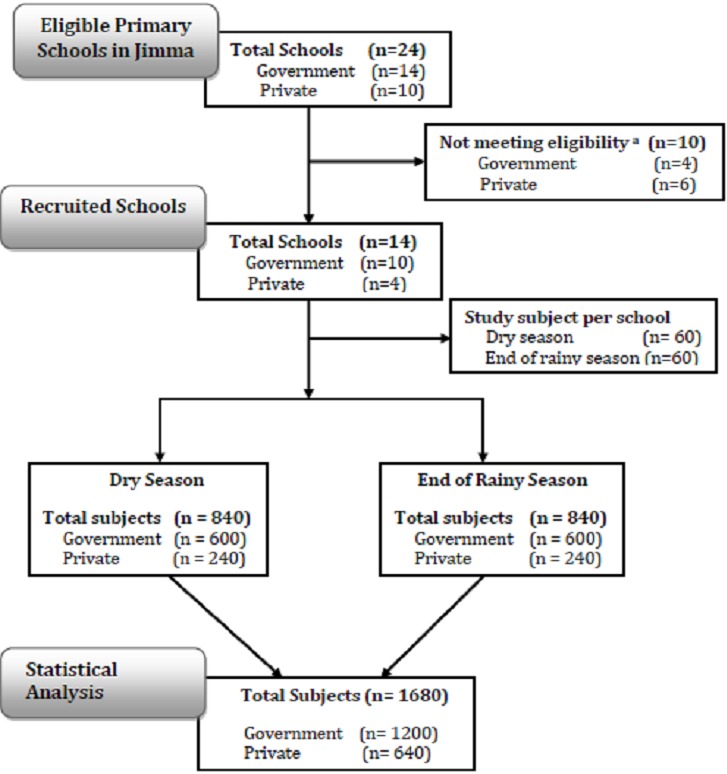
Flow chart illustrating number of schools and stool samples for assessing the seasonality of infection intensity and prevalence of STHs in Jimma (Ethiopia). Eligibility criteria for schools was hosting grade 1-8 students (age from 5-18 years) and/or provision of < 60 samples per school per season

**Study design:** The assessment of infection intensity and prevalence of STHs was carried out during dry season (February-March, 2012) and end of rainy season (September-October, 2012) among 14 schools in Jimma, Ethiopia. For this study, a total of 1,680 SAC (840 in each season) were included. Sample size was estimated based on varying epidemiological scenarios that at least 120 subjects per school (60 per season per school) were required for a reliable estimate of apparent prevalence and infection intensity at school level. To this end, all primary schools in Jimma Town hosting all eight grades of students were invited to participate. In each school subjects were stratified according to three age classes (age class A: age 5-9 years, B: age 10-13 years and C: age 14-18 years). For each age class at least 20 subjects were selected on a voluntary basis, resulting in a total of at least 60 subjects per school. The subjects were asked to provide at least 3 g of stool. This quantity of stool was required to examine the samples individually (2 g) after processed with the McMaster egg counting method (analytic sensitivity of 50 eggs per gram of stool (EPG)) for detection and enumeration of STH eggs [16]. Figure 2 illustrates the number of primary schools eligible, recruited, and included in the statistical analysis.

**Parasitological examination:** All stool samples were processed by the McMaster egg counting method as previously described by Levecke *et al*, 2011 [16]. In short, two grams of stool was suspended in 30 ml of saturated salt solution. The faecal suspension was poured three times through a wire mesh to remove large debris. Then, 2 chambers of McMaster slide were filled with this suspension. Both chambers were examined under a light microscope using a 100x magnification and faecal egg count (FEC) as eggs per gram of stool (EPG) for each helminth species were obtained by multiplying the total number of eggs by 50. A tutorial for stool sample processing and performing the McMaster egg counting method is made available at: http://www.youtube.com/watch'v=UZ8tzswA3tc.

**Statistical analysis:** The prevalence (% of children excreting eggs) and the infection intensity (mean FEC) were calculated on STH species for the different schools, both sexes, three age classes (A: 5-9 years; B: 10-13 years and C: 14-18 years) and two seasons (dry vs. rainy). Odds ratio was computed to assess any differences in prevalence and infection intensity between the two seasons. To this end, random effect models were built at the school level based on the odds ratio and the difference in mean FEC (mean FEC in the rainy season-mean FEC in the dry season), respectively. The statistical analysis was performed in the statistical software R (The R Foundation for Statistical Computing, version 3.0). The pooled odd ratio was also computed and presented using the 'metafor' package of the statistical software R. The level of significance was set at p <0.05.

## Results

**Prevalence and infection intensity:** **Table 1** reports the prevalence and infection intensity of any STHs and three species separately for the schools, two sexes, three age classes and two seasons. Infections with any STHs were observed in 824 out of the 1,680 subjects screened (49.0%). *T. trichiura* was the most prevalent (n = 596, 35.5%), followed by *A. lumbricoides* (n = 394, 23.4%). Hookworm was observed in 166 subjects (9.9%). STH infections were observed in all 14 schools, except for hookworms for which infections were absent in 2 schools. Among the schools there was a large variation in prevalence, ranging from 16.7% to 68.3% for any STH, from 6.7% to 39.2% for *A. lumbricoides*, from 10.8% to 55.0% for *T. trichiura* and from 0 % to 28.3% for hookworms. Variation in infections between sexes (females vs. males) was small both for any STH (50.0% vs. 48.2%) and the three STH species separately (*A. lumbricoides*: 23.3% vs. 23.6%; *T. trichiura*: 34.5% vs. 36.3%; hookworm: 10.1% vs. 9.7%). There was increase in STH infections across age, with the proportion of infections increasing from 47.7% for age class A to 52.0% to age class C. An increase across age classes was also observed for hookworms (A: 5.8%; B: 10.1%; C: 13.8%). For *T. trichiura* a decrease in prevalence across age classes was observed (A: 37.1%; B: 35.2%; C: 34.2%). For *A. lumbricoides* no trend was observed (A: 26.0%; B: 21.2%; C: 23.4%). STH infections were more prevalent in the dry season (52.4%) compared to the rainy season (45.7%). This seasonality in prevalence was most pronounced for *T. trichiura* (39.2% vs. 31.8%), followed by hookworms (11.4% vs. 8.3%). For *A. lumbricoides*, the proportion of infections across seasons was comparable (23.8% vs. 23.0%). Overall, the mean FEC was 1,938 EPG, 207 EPG and 29 EPG for *A. lumbricoides*, *T. trichiura* and hookworm, respectively. Among the schools there were large variation in FEC for each of the three STH, ranging from 317 EPG to 4,926 EPG for *A. lumbricoides*, from 18 EPG to 532 EPG for *T. trichiura* and from 0 EPG to 60 EPG for hookworms. Variation in infections between sexes (females vs. males) was small (*A. lumbricoides*: 1,908 EPG vs. 1,968 EPG; *T. trichiura*: 199 EPG vs. 214 EPG; hookworm: 31 EPG vs. 28). There was decrease in infection intensity across age for both *A. lumbricoides* (A: 2,884 EPG; B: 1,727 EPG; C: 1,228 EPG) and *T. trichiura* (A: 305 EPG; B: 177 EPG; C: 143 EPG). However, an increase across age classes was observed for hookworms (A: 17 EPG; B: 31 EPG; C: 40 EPG). Intensity of all three STH infections was higher in the dry season. This seasonality in infection intensity was most pronounced for *A. lumbricoides* (2,411 EPG vs. 1,465 EPG), followed by *T. trichiura* (295 EPG vs. 119 EPG) and hookworms (35 EPG vs. 23 EPG).

**Table 1:** The prevalence and infection intensity by means of faecal egg counts (FEC) across 14 schools in Jimma Town, Ethiopia, 2 sexes, 3 age classes and 2 seasons

	N	STH (%)	*A. lumbricoides*		*T. trichiura*		Hookworms	
			Prevalence (%)	Mean FEC (EPG)	Prevalence (%)	Mean FEC (EPG)	Prevalence (%)	Mean FEC (EPG)
**School**								
1	120	29.2	12.5	555	20.0	63	0	0
2	120	16.7	6.7	436	10.8	18	0.8	1
3	120	57.5	30.0	1,983	40.8	192	9.2	42
4	119	26.9	6.7	317	26.1	77	0.8	5
5	120	62.5	35.8	4,138	42.5	334	20.8	60
6	120	65.0	27.5	2,055	52.5	532	5.8	37
7	120	54.2	22.5	2,840	37.5	218	12.5	52
8	120	68.3	39.2	3,242	55.0	262	6.7	10
9	120	60.0	33.3	1,875	34.2	315	28.3	50
10	120	60.0	33.3	4,926	41.7	225	15.8	43
11	120	51.7	26.7	1,892	38.3	290	7.5	25
12	120	55.0	17.5	705	41.7	158	12.5	35
13	121	55.4	28.9	1,550	34.7	143	17.4	51
14	120	24.2	7.5	612	20.8	72	0	0
**Sex**								
Female	782	50.0	23.3	1,908	34.5	199	10.1	31
Male	898	48.2	23.6	1,968	36.3	214	9.7	28
**Age Class**								
A	539	47.7	26.0	2,884	37.1	305	5.8	17
B	603	47.6	21.2	1,727	35.2	177	10.1	31
C	538	52.0	23.4	1,228	34.2	143	13.8	40
**Season**								
Dry	840	52.4	23.8	2,411	39.2	295	11.4	35
Rainy	840	45.7	23.1	1,465	31.8	119	8.3	23
**Total**	**1,680**	**49.0**	**23.5**	**1,938**	**35.5**	**207**	**9.9**	**29**

14 school; 2 sexes; 3 age classes; and 2 seasons

**Figure 3:**
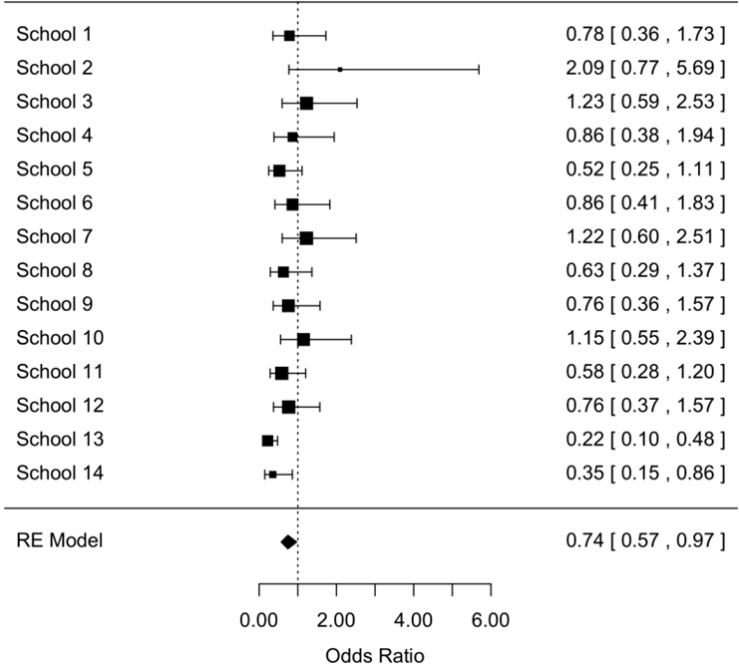
The odds of any STH infection during dry season and rainy season (95% confidence interval)

**Figure 4:**
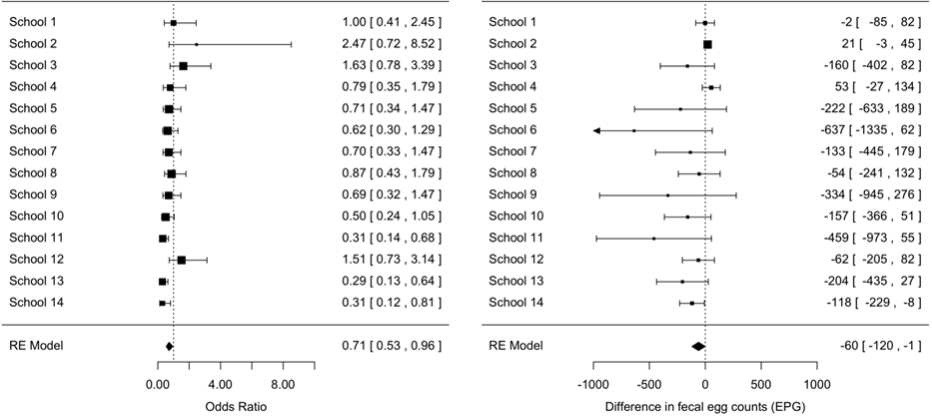
Significant difference in *Trichuris trichiura* prevalence and infection intensity across seasons

**Figure 5:**
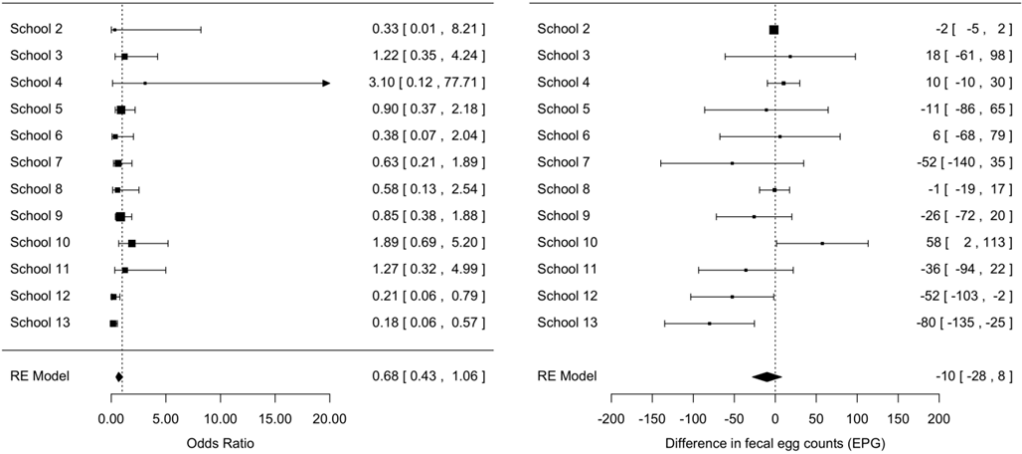
Difference in hookworms' prevalence across seasons and infection intensity across seasons

**Figure 6:**
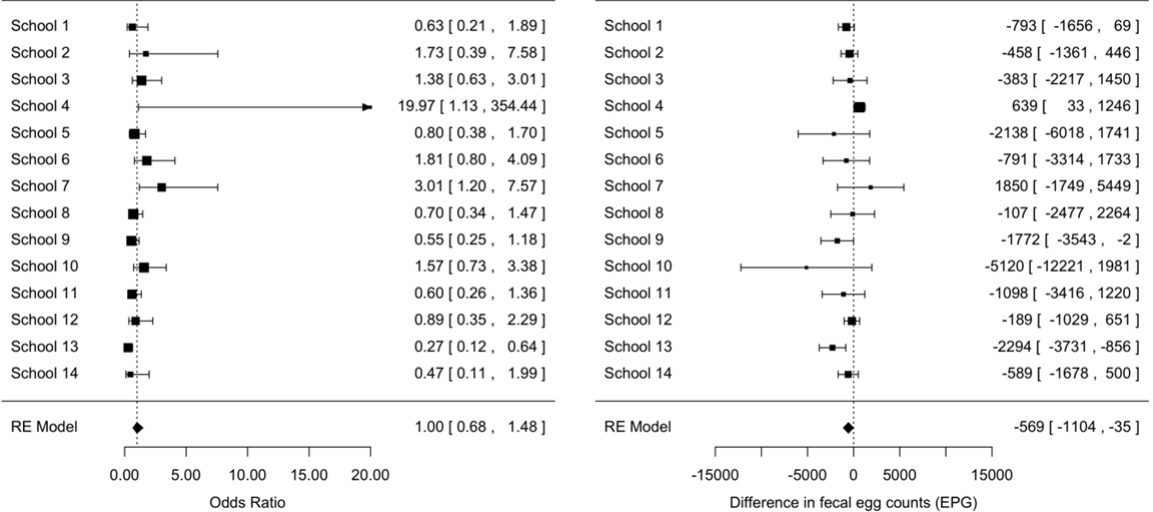
The odds ratio *A. lumbricoides* infections across the seasons

**Seasonal differences in prevalence and infection intensity:** Overall, any STH infection was less prevalent in the rainy season compared to the dry season, the odds of any STH infection being 0.74 (95% confidence interval [0.57-0.97], z = -2.19, p = 0.03) times smaller than the odds of any STH infection in the dry season (Figure 3). A significant difference in prevalence across seasons for the three STH species separately was only observed for *T. trichiura infections* (Figure 4). For this STH, the odds of infection in the rainy season were 0.71 ([0.53-0.96], z = -2.26, p = 0.02) times smaller than those in the dry season. A comparable odds ratio (0.68 [0.68-1.06]) was observed for hookworms, however this ratio was not significantly different from 1 (z = -1.71, p = 0.06) (Figure 5). For *A. lumbricoides* infections the odds ratio equalled 1.00 [0.68-1.48] (z = 0.02, p = 0.98) (Figure 6). A significant difference in infection intensity across seasons was observed for *A. lumbricoides* (Figure 6) and *T. trichiura* (Figure 4). For both STH species, infection intensities were lower in the rainy season compared to those in the dry season. The estimated difference infection intensity equalled 569 EPG ([35-1104], z = -2.09, p = 0.04) for *A. lumbricoides* and 60 EPG ([1-120], z = -1.98, p = 0.05) for *T. trichiura*. For hookworms, estimated difference was 10 EPG ([-8-28], z = -1.11, p = 0.27) no significant difference in infection intensity across seasons was observed.

## Discussion

Overall, the prevalence and intensity of STH infection is high among SAC in Jimma Town where nearly half of the children are infected at least with one STH species. Our data showed even within a defined geographic area located in less than 10 km radius (Figure 1), huge variation was observed in any STHs prevalence and intensity. This is in line with studies who have revealed that STHs show considerable and distinct spatial heterogeneity [17-19]. This finding is supported with the argument that the precise distributions of STH infection and their underlying demographic, socio-economic and environmental determinants, still remains poorly defined. It has been documented that unhygienic sanitation, inadequate water supply and the use of untreated night soil fertilizer are man-made environmental factors that are particularly favorable for transmission of these helminths [6, 20]. Yet the ecological and epidemiological features that might explain the spatial heterogeneity of STHs distribution among those schools in Jimma Town, however, remain to be investigated. Specially consideration should be placed on the geological composition and soil types of the specific study locations given that these environmental factors have been shown to play significant role in the transmission of STH [21-25], which is the limitation of this study, as we did not explore those factors.

Compared to end of rainy seasons (September-October) SAC were shown to have marginally higher prevalence of both *T. trichiura* and hookworms, but relatively uniform *A. lumbricoides* infections during dry seasons (January-February). In the same line, Nwoke *et al*, [25] have reported highest (18.7%) recovery of parasites including STHs from soil in dry season as compared to low recovery rate (12.0%) in wet seasons, indicating seasonal variation of soil contamination level by STHs. However, the present findings disagree with the findings of previous studies reported elsewhere [12, 13] that showed highest prevalence of STH in rainy season compare to dry season. Looking at infection intensity across age classes, the observed trend revealed a concentrated EPG in the age class A (5-9 years) for both *A. lumbricoides* and *T. trichiura* and tend to decline across age class B (10-13 years) and C (14-18 years), which is consistent with findings of previous studies elsewhere [26-28]. On the other hand, a significant difference in infection intensity across seasons was observed for both *A. lumbricoides* and *T. trichiura* but not hookworms during dry season. This significantly higher intensity of *T. trichiura* and *A. lumbricoides* during the dry season probably attributed to a favourable and conducive conditions created by different seasons for transmission for those parasites. In particular for *T. trichiura*, it appeared it is more affected by the large temporal changes in the environment in comparison to hookworms and/or *A. lumbricoides*. Nevertheless, it has been argued that although seasonal dynamics in transmission may occur, such fluctuations may be of little significance to the overall parasite equilibrium within communities [23, 28, 29].

Taken as a whole, understanding where at-risk populations live including the most risk full season for infection/dynamic transmission and to what extent people are intensively infected, is fundamental for appropriate resource allocation and cost-effective control. In particular, it allows for reliable estimation of the overall drug needs of programmes, frequency of treatment and efficient geographical/specific areas targeting of control efforts [9, 29]. On the other hand, failing to understand all those factors and the anticipated rapid re-infection of STHs might limits how national governments and international organizations define and target resources to combat the disease burden due to STH infection through PC programmes.

## Conclusion

We conclude that STH infections are a public health problem in Jimma, but prevalence and infection intensities vary considerably among schools and between seasons, calling for urgent control programme (PC) complemented by improved access to clean water and adequate sanitation, coupled with sound health education. Moreover, long term studies are needed to confirm the influence of seasonal factors and related ecological, environmental and socio-economic factors on prevalence and intensity of STH infections in SAC. However, this study suggest climate change leading to potentially larger seasonal fluctuations of the environment may affect patterns and intensity of STH infections and further exacerbate health impacts on SAC and other risk groups that live in highly variable seasonal environments.

### What is known about this topic

- Prevalence of STH is the only indicator that guides the frequency of preventive chemotherapy;
- The main stay strategy currently underway to control STHs infection is mass drug administration with either albendazole or mebendazole as PC;
- Evidence indicated that socio-economic, environmental and behavioural factors are related to the prevalence and incidence of STH infections.

### What this study adds

- The presence of overall high prevalence of STH in the study area indicating it is a public health importance;
- The presence of huge variation (both in prevalence and intensity) of STH species among schools even within limited geographical location indicating the magnitude of the challenges for control programmes;
- Seasonal fluctuations of the environment may affect patterns and intensity of STH.

## Competing interests

The authors declare no competing interests.
